# Involvement of *PaSNF1* in Fungal Development, Sterigmatocystin Biosynthesis, and Lignocellulosic Degradation in the Filamentous Fungus *Podospora anserina*

**DOI:** 10.3389/fmicb.2020.01038

**Published:** 2020-06-10

**Authors:** Yuanjing Li, Pengfei Yan, Xiaojie Lu, Yanling Qiu, Shang Liang, Gang Liu, Shuangfei Li, Lin Mou, Ning Xie

**Affiliations:** ^1^Shenzhen Key Laboratory of Microbial Genetic Engineering, College of Life Sciences and Oceanography, Shenzhen University, Shenzhen, China; ^2^Key Laboratory of Functional Inorganic Material Chemistry (MOE), School of Chemistry and Materials Science, Heilongjiang University, Harbin, China

**Keywords:** *Podospora anserina*, sucrose non-fermenting 1, sexual development, stress tolerance, secondary metabolism, lignocellulose degradation

## Abstract

The sucrose non-fermenting 1/AMP-activated protein kinase (SNF1/AMPK) is a central regulator of carbon metabolism and energy production in the eukaryotes. In this study, the functions of the *Podospora anserina* SNF1 (PaSNF1) ortholog were investigated. The Δ*PaSNF1* mutant displays a delayed development of mycelium and fruiting bodies and fails to form ascospores. The expression of the *PaSNF1* gene in the strain providing female organs in a cross is sufficient to ensure fertility, indicating a maternal effect. Results of environmental stress showed that Δ*PaSNF1* was hypersensitive to stress, such as osmotic pressure and heat shock, and resistant to fluconazole. Interestingly, the knockout of *PaSNF1* significantly promoted sterigmatocystin (ST) synthesis but suppressed cellulase [filter paperase (FPA), endoglucanase (EG), and β-glucosidase (BG)] activity. Further, transcriptome analysis indicated that *PaSNF1* made positive regulatory effects on the expression of genes encoding cellulolytic enzymes. These results suggested that *PaSNF1* may function in balancing the operation of primary and secondary metabolism. This study suggested that SNF1 was a key regulator concerting vegetative growth, sexual development, and stress tolerance. Our study provided the first genetic evidence that SNF1 was involved in the ST biosynthesis and that it may also be a major actor of lignocellulose degradation in *P. anserina*.

## Introduction

Lignocellulose is one of the most abundant renewable resources on earth, mainly composed of cellulose, hemicellulose, and lignin (Jonsson et al., 2013). It can be converted into different carbohydrates, including glucose, xylose, and aromatic monomer compounds, which could be applied for the production of ethanol, carbohydrates, and aromatic products (Ragauskas et al., 2014; Kawaguchi et al., 2016; Wu et al., 2018). It has promising applications in food, paper, and textile industries as well as the production of biofuels and other chemicals (Paulova et al., 2015; Kubicek and Kubicek, 2016; Yenkie et al., 2016). However, the commercial applications of lignocellulosic degradation in the production of biofuels and chemicals were limited by its complex structure, biological obstinacy, low efficiency, and high cost, thus hindering the effective utilization of the raw material in green technology (Weng et al., 2008; Paulova et al., 2015). Biodegradation of lignocellulose into fermentable sugars with cellulase [including Carbohydrate-Active Enzymes (CAZy)] has been an environmental-friendly approach (Gupta et al., 2016). Filamentous fungi were considered as promising candidates for producing lignocellulolytic enzymes (Couturier et al., 2016).

Filamentous fungi and their enzymes show great potential in the degradation of tough lignocellulose (Bomble et al., 2017). Some ascomycetes such as *Trichoderma reesei* and *Aspergillus nidulans* have been commonly applied in the industrial production of cellulase and hemicellulase (de Assis et al., 2015; Bischof et al., 2016). Many cellulose and lignin degradation-associated enzymes were found in the filamentous fungus *Podospora anserina*, making this fungus a model to elucidate the enzymatic decomposition of plant biomass (Couturier et al., 2016). Recent studies have shown that catalase, laccase, and bilirubin oxidase participated in the degradation of cellulose in *P. anserina* (Bourdais et al., 2012; Xie et al., 2014, 2015). The regulation mechanism of cellulase and hemicellulase gene expression has been extensively investigated (Benocci et al., 2017). In cellulolytic fungi, several key transcriptional activators as well as repressors have being involved in this regulation. For example, the transcription factor XlnR/XYR1 regulated the expression of cellulase and hemicellulase genes in *Aspergillus niger*, *Neurospora crassa*, and *T. reesei*, respectively (Stricker et al., 2008; Sun et al., 2012; Dos Santos, Castro et al., 2016). In addition, the transcription factors CLR-1 and CLR-2 were essential for growth on cellulose in *N. crassa* (Coradetti et al., 2012). Recent studies have shown that the CLR-4 acted as a positive activator of cellulase expression by directly binding to the promoters of CLR-1 and CLR-2 (Liu et al., 2019). CLR-3 repressed the cellulolytic response through CLR-1 (Huberman et al., 2017). The conserved zinc-finger transcription factor Cre1/CreA was the key factor in mediating carbon catabolite repression (CCR), an important mechanism for suppressing cellulase production during growth on favorably metabolized carbon source, such as glucose (Sun and Glass, 2011; Lichius et al., 2014). Deletion of Cre1/CreA alleviated some aspects of CCR for cellulolytic enzyme expression in *Penicillium decumbens* and *N. crassa* (Sun and Glass, 2011; Liu et al., 2013). Therefore, transcriptional activation or de-repression via nutrient sensing pathways has also been essential for inducing the lignocellulolytic response.

Sucrose non-fermenting 1/AMP-activated protein kinase (SNF1/AMPK kinases) was regarded as an important regulator that participated in the development of fungi and activation of catabolite-repressed genes (Lee et al., 2009; Ghillebert et al., 2011). In yeast, as a heterotrimer kinase complex, SNF1 contained α-subunit encoded by *SNF1*; β-subunits encoded by *GAL83*, *SIP1*, and *SIP2*; and γ-subunit encoded by *SNF4*. The SNF1 complex can be activated by three upstream kinases (Sak1, Elm1, and Tos3) via phosphorylation (Hedbacker and Carlson, 2008), whereas it could also be inactivated by Reg1-Glc7 protein phosphatase (Sanz et al., 2000). The regulation of SNF1 activity played a central role in the alleviation of CCR, for both *Saccharomyces cerevisiae* and the majority of filamentous fungi (Brown et al., 2013). In yeast, under the glucose starvation, Mig1 was phosphorylated by SNF1, inducing the de-repression of glucose repressed genes. Studies have shown that the CCR mechanism was different in filamentous fungi and yeast. CreA, a Mig1 homolog, was a critical regulator of carbon catabolism inhibition in the lignocellulolytic fungus *A. nidulans* and the orthologous SNF1 kinase was essential for CreA/Cre1 de-repression (Brown et al., 2013). However, homologous proteins of Cre1 could not be phosphorylated by homologous proteins of SNF1 in *T. reesei* (Cziferszky et al., 2003). Overall, it was unclear whether there was a conserved mechanism for *SNF1* to regulate carbon catabolite in filamentous fungi. Little was known about the functions of *SNF1* in the fungal development and lignocellulosic degradation.

Besides, SNF1 complex participated in a wide variety of physiological processes, such as fungal meiosis, spore formation, filamentous growth, and biofilm formation (Lee et al., 2009; Ghillebert et al., 2011). It was also involved in the response to environmental stresses, such as salt stress, heat shock, alkaline pH, and endoplasmic reticulum stress (Hahn and Thiele, 2004; Hong and Carlson, 2007; Casamayor et al., 2012; Ferrer-Dalmau et al., 2015). For many pathogenic filamentous fungi, *SNF1* genes showed critical roles in fungal development and virulence, such as *Magnaporthe oryzae* (Yi et al., 2008), *Gibberella zeae* (Lee et al., 2009), *Verticillium dahlia* (Tzima et al., 2011), *Penicillium digitatum* (Zhang et al., 2013), *Beauveria bassiana* (Wang et al., 2014), *Leptosphaeria maculans* (Feng et al., 2014), and *Fusarium virguliforme* (Islam et al., 2017). The lack of *SNF1* led to inhibitory expression of cell-wall-degrading enzymes, damage of sporulation, and spore germination; thus, its virulence on the hosts would be impaired.

Biosynthesis of secondary metabolites (SMs) may be tightly regulated. Their expression was often triggered by chemical or environmental stimuli and coordinated with the development and morphogenesis of the producing organism. Sterigmatocystin (ST) was one of the ultimate precursors in the biosynthesis of the aflatoxins. The gene cluster responsible for the ST biosynthesis was identified in *A. nidulans* by Brown et al. (1996). A similar cluster of genes was found in *P. anserina* (Slot and Rokas, 2011). ST was detected in cultures of a freshly isolated *P. anserina* strain (Bills and Gloer, 2016). The ST biosynthetic gene cluster was regulated by the cluster-specific AflR transcription factor, which functioned downstream of glucose sensing (Fernandes et al., 1998). Overexpression of *PaAflR* led to a ST overproduction and a female sterility phenotype in *P. anserina* (Shen et al., 2019). Additionally, LaeA also regulated the ST biosynthetic gene cluster in an AflR-dependent manner. The Δ*LaeA* mutation abolishes AflR expression and subsequently the production of ST and other SMs (Bok and Keller, 2004). Interestingly, Cre1, which regulated cellulase production, was also involved in the synthesis of SMs (Monroy et al., 2017). However, the importance of *SNF1* and their functional connections in regulating SMs have been unknown.

Elucidating the physiological functions of *SNF1* contributed to understanding the mechanisms of filamentous fungi sense nutrition and cellular energetic status, which was paramount for the industrial production of cellulase. In this study, we identified a homolog of *SNF1* in the genome of *P. anserina*. With the use of a homologous recombination strategy, the disruption mutant of *P. anserina* SNF1 (*PaSNF1*) was constructed, and the effects of *PaSNF1* in fungal development and lignocellulosic degradation were characterized. Δ*PaSNF1* was characterized for defects in sexual reproduction (e.g., fruiting body formation), colony formation, ascospore germination, and stress responses. Our study provided the first genetic evidences that *SNF1* was involved in the ST biosynthesis. Enzymology and transcriptome analysis confirmed that *PaSNF1* played important roles in cellulose degradation.

## Materials and Methods

### Strains and Culture Conditions

All strains of *Podospora anserina* applied in this study were derived from the “S” wild-type (WT) strain, which was sequenced for obtaining the genome of the *P. anserina* (Espagne et al., 2008; Grognet et al., 2014; Silar et al., 2019). The standard culture protocol of *P. anserina* could be found at http://podospora.igmors.u-psud.fr/methods.php. The Δ*mus51:Hygro* mutant strain differed from the S WT reference strain by a single deletion of the *mus-51* gene, in which frequency of targeted gene replacement was increased (El-Khoury et al., 2008).

### Phylogenetic Analysis

The putative SNF1 genes of *P. anserina* were identified by BLAST at the *P. anserina* website by retrieving various fungal SNF1^1^. In this study, the putative SNF1 encoded by *Pa_2_770* was named as PaSNF1. The alignment was manually refined, and sites with ambiguous alignment were removed from the dataset. The phylogenetic tree was reconstructed using a neighbor-joining method, and an alignment was produced with the CLUSTALW algorithm in the MEGA5 software (Wang et al., 2014), in which the numbers at nodes indicated bootstrap values with 1,000 replications.

### Generation of Deletion Mutants and Complementation

A deletion cassette for the *SNF1* (*Pa_2_770*) gene was constructed with a geneticin resistance cassette as previously reported (Xie et al., 2014, 2015, 2018). The Δ*mus51:Hygro* protoplasts were transformed by the PCR products with geneticin cassette. Transformants were screened on selective medium containing 100 μg/ml of geneticin and 75 μg/ml of hygromycin. Putative deleted mutants were firstly selected by PCR. The PCR amplified specific junctions to the replaced locus, with two primers that annealed at one end of the selectable resistance gene and upstream of the proximal flank in the deletion cassette (Supplementary Table S1). Homologous recombination of the deletion cassette allowed amplification of a predictable fragment on each side of the selectable resistance gene. Three candidate transformants would be genetically purified by crossing them with WT. It aimed to eliminate the potential untransformed nuclei and then segregate out the Δ*mus-51* mutation. Mat- and mat + geneticin-R and hygromycin-S strains were identified from each progeny, and one transformant was subjected to Southern blot analysis for final validation (Supplementary Figure S2). Only purified transformants verified by Southern blot would be selected as stock deletion mutants for subsequent studies.

To ensure that the relevant *PaSNF1* gene was inactivated in the derived phenotypes, the complementation procedure was done by ectopic insertion of corresponding gene under the control of its native promoter. The WT with complete CDS and the 5′ (500 bp) and 3′ (200 bp) flanking regions were amplified by PCR using a high-fidelity polymerase and primers of PaSNF1-cF and PaSNF1-cR (Supplementary Table S1). The DNA fragment was cloned into the pBC-Nourseo (Lalucque et al., 2012) at the *Not*I and *Bam*HI sites to yield pBC-Nours-*PaSNF1* plasmid. This plasmid was transformed into the protoplasts of the Δ*PaSNF1* mutant strain. Transformants (CP*PaSNF1*) were screened on selective medium with 40 μg/ml of nourseothricin (Xie et al., 2018). The presence of the WT allele was also verified by PCR.

### Growth and Development in Standard Conditions

To determine the roles of *PaSNF1* in fungal physiology, both the deleted mutant and WT were incubated separately on M_2_ medium at 27°C. Colony morphology, pigmentation, perithecium formation, ascospore production, ascospore dispersal, and germination were observed during the vegetative growth and sexual cycle. Three plates were set for each treatment, and the experiment was repeated once.

### Stress Sensitivity Assays

In this study, mat + mating type strains were used unless specified. For evaluating the stress sensitivity, WT, Δ*PaSNF1*, and CP*PaSNF1* were incubated separately on M_2_ medium, supplemented with osmotic stress reagents (0.5 M of NaCl, 0.5 M of KCl, 0.5 M of sorbitol, and 0.75 M of glycerol), oxidative reagents (0.01% H_2_O_2_ and 50 μM of menadione), cell wall inhibitors [0.02% Congo red and 0.05% sodium dodecyl sulfate (SDS)], and cell membrane inhibitor (2.5 μg/ml of fluconazole). The menadione was obtained from Macklin, and fluconazole was purchased from Aladdin. Fresh mycelium was incubated at 45°C in water bath for 2 h. Equal volume of mycelium was inoculated on M2. Equal volume of mycelium without heat shock served as control. All cultures were incubated at 27°C for 3 days, colony size was measured, three plates were set for each treatment, and the experiment was repeated once.

### Detection of Secondary Metabolites by High-Performance Liquid Chromatography

To analyze the SMs, both WT and Δ*PaSNF1* (mat + mating type) strains were incubated separately in 100 ml of liquid medium (in 250-ml triangle bottle) at 27°C, shaking at 150 rpm for 7 days. Mycelium and the liquid phase were separated by centrifugation. The supernatant was extracted for three times with an equal volume of ethyl acetate. The organic phase was transferred for evaporation to yield a concentrated residue. The residue was re-suspended in 1 ml of methanol and then centrifuged at 12,000 rpm for 10 min. The obtained supernatant was filtered with 0.22-μm Millipore filter and then analyzed with high-performance liquid chromatography (HPLC) (Waters e2695 separations Module) with 2998 PDA detector with ultimate XB-C18 column (4.6 × 250 mm, Welch Materials, Inc.). The conditions for HPLC were as follows: flow rate was 1 ml/min, column temperature was 30°C, and the solvent gradients were solvent A (H_2_O) and solvent B (methanol). The sample was detected for 20 min using a linear gradient of 10–90% (0–20 min), 100% MeOH (20.01–30 min), and 10% MeOH (30.01–30 min). The wavelength of UV detector was set at 254 nm.

### Characterization of Sterigmatocystin Synthesis in Δ*PaSNF1* Mutant

For the purification of these compounds, Δ*PaSNF1* mutant (mat + mating type) were cultured in 5 l of liquid medium at 27°C, shaken at 150 rpm for 7 days. Culture media were Solution 1 0.5% (v/v), Solution 2 0.5% (v/v), Solution 3 0.5% (v/v), Solution 4 0.5% (v/v), biotin 0.05% (v/v), thiamine 0.05% (v/v), microelement 0.1% (v/v), yeast extract 0.5% (m/v), and dextrin blonde 0.55% (m/v). The standard culture protocol of *P. anserina* could be found at http://podospora.igmors.u-psud.fr/methods.php. The culture was extracted with ethyl acetate for three times. The organic phase was evaporated to obtain the crude residue (1.3 g). The residue was separated on silica gel column with a stepped gradient elution of CH_2_Cl_2_–MeOH (100:0–0:100) to yield 6 subfractions (Fr. 1–Fr. 6). Fr. 1 (a total of 105.4 mg) was further purified by semi-preparative HPLC, and ST was gained (at 18.5 min). Nuclear magnetic resonance (NMR) spectra were performed on a Bruker Avance III 500 spectrometer (600 MHz). DMSO-*d*_6_ was applied as the solvent for analyzing ST (Pachler et al., 1976; Oleinikova et al., 2012).

### Cellulose Degradation and Enzyme Assays

For evaluating the activity of cellulase, both WT and Δ*PaSNF1* (mat + mating type) were cultured. The mycelium agar pieces (2 × 2 mm) from actively growing WT and mutant strains were transferred to Petri dishes with cellophane placed on M2 medium, and then the mycelia were incubated at 27°C for 2 days (Xie et al., 2015). The harvested mycelium was smashed in 200 μl of extraction buffer (sodium citrate buffer 50 mM, pH7.0) by FastPrep (4.0) for 20 s. All the samples were centrifuged at 10,000 rpm for 10 min to obtain the supernatants. The activity of FPA and EG were measured by the 3,5-dinitrosalicylic acid (DNS) method with filter paper and carboxymethyl cellulose as substrates (Liao et al., 2018). The activity of BG and exo-1,4-β-glucanase (CBH) was determined using *p*-nitrophenyl-β-D-glucopyranoside (pNPG) and *p*-nitrophenol-D-cellobioside (pNPC) as the substrates, respectively (Li et al., 2016). Protein concentration was quantified with Bradford method (Bradford, 1976). All the determinations were performed in triplicate. To evaluate the cellulose degradation, the M2 standard minimal synthetic medium contains dextrin (0.5%) as carbon source. Dextrins were replaced with microcrystalline cellulose (MCC; 0.5%) from Sigma and 0.5 g/plate straw materials. Fragmented mycelia from mat + and mat- strains of WT or Δ*PaSNF1* were mixed and then inoculated in the center of the plate under constant light illumination. Photographs were taken after 7 days of incubation.

### Transcriptome Analysis

The mycelia of WT and Δ*PaSNF1* grown in cellulose (3 days) were harvested by filtration and immediately frozen in liquid nitrogen. The culture medium was a fungal liquid culture medium. An equivalent amount of carbon source dextrin was replaced with microcrystalline cellulose. The composition of the medium was as follows: Solution 1 0.5% (v/v), Solution 2 0.5% (v/v), Solution 3 0.5% (v/v), Solution 4 0.5% (v/v), biotin 0.05% (v/v), thiamine 0.05% (v/v), microelement 0.1% (v/v), yeast extract 0.5% (m/v), and Avicel (Sigma) 0.55% (m/v). Total RNA was extracted using TRIzol^®^ RNA reagent (Invitrogen, Carlsbad, CA, United States) and further purified using DNase I (RNeasy Mini kit, Qiagen, Hilden, Germany). NanoDrop and agarose gel electrophoresis were used to check RNA integrity and concentration.

Qualified RNA with an OD260/OD280 > 1.8 and RNA integrity number (RIN) > 7.0 was prepared according to Majorbio (Shanghai, China) standard protocols and sequenced on an Illumina HiSeq 2000/2500 platform (San Diego, CA, United States). All data in this study were generated by sequencing two independent duplicate samples. Total clean reads were mapped against predicted transcripts from the WT *P. anserina* strain genome^2,^^3^ to assess sequence homology and functional annotation. Read mapping and counting using TopHat (version 2.1.1) and hisat2 (version 2.1.0) were performed. RSEM (version 1.3.1) software was used to analyze gene expression levels with the fragments per kilobase of exon per million mapped reads (FPKM) method. Differentially expressed genes (DEGs) were screened using the DESeq2 (version 1.24.0) with fold change of more than 1.5 and *p*-value ≤ 0.05 as thresholds. The dataset was also manually screened for the genes encoding carbohydrate active enzymes. Hierarchical clustering analysis was performed using the pheatmap package in R (version 3.6.1)^2^. RNA-Seq data were deposited in the National Center for Biotechnology Information (NCBI) Sequence Read Archive (SRA) under accession number PRJNA597840.

### Quantitative RT-PCR

The mycelia of WT and Δ*PaSNF1* grown in cellulose (3 days) were harvested by filtration and immediately frozen in liquid nitrogen. Total RNA was extracted using TRIzol^®^ RNA reagent (Invitrogen, Carlsbad, CA, United States) for total RNA extraction. All total RNA samples were reversely transcribed into cDNAs and then assessed for the transcript levels of 20 genes via RT-qPCR with paired primers (Supplementary Table S1). All RT-qPCR experiments with three cDNA samples of each strain were performed under the action of SYBR^®^ Premix Ex TaqTM (TaKaRa, Dalian, China). Initially, three reference genes were used for normalization: *GPD* (glyceraldehyde 3-phosphate dehydrogenase, formally *Pa_3_5110*), *PDF2* (protein phosphatase PP2A regulatory subunit A, *Pa_7_6690*), and *UBC* (ubiquitin, *Pa_4_7790*). All three were stably expressed; comparatively speaking, *PDF2* has the best stability, and in RNA-Seq data, the difference between the FPKM of *PaPDF2* in WT and Δ*PaSNF1* was not significant. Thus, only the *PaPDF2* gene was used as a reference gene to normalize gene expression results. The ratio of each gene transcript in Δ*PaSNF1* over that in WT was converted to log2 for comparison with the corresponding log2 ratio from the RNA-Seq data.

### Microscopic Observations

The perithecia of mycelia grown on agar were observed and captured with a homemade microscope, combining a camera [monochromatic 1/3′′ complementary metal–oxide–semiconductor (CMOS) sensor with a resolution of 1,280 × 960 pixels] and a telecentric objective (4 × magnification and 65-mm focal length). Pictures of squashed perithecia and ascospore were captured with a Nikon eclipse Ni-V microscope and analyzed with ImageJ.

## Results

### Identification of SNF1 Homolog in *Podospora anserina* and Phylogenetic Analysis

**FIGURE 1:**
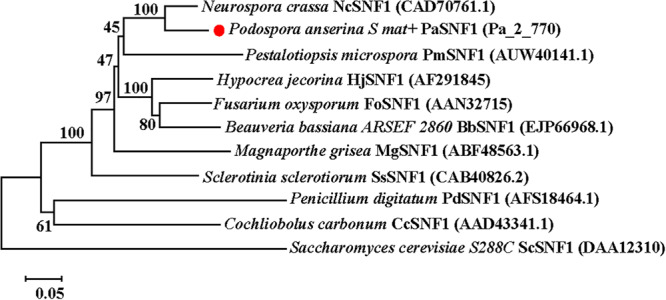
Phylogenetic analysis of *PaSNF1* and SNF1 orthologs identified in other organisms. The phylogenetic tree was reconstructed using a neighbor-joining method. Numbers at nodes indicate bootstrap values with 1,000 replications. GenBank accession number of a given SNF1-coding gene was parenthesized following the name of each fungal species. Distance scale: scale that represents the number of differences between sequences (e.g., 0.05 means 5% differences between two sequences). SNF1, sucrose non-fermenting 1; *PaSNF1*, *Podospora anserina* SNF1.

The genome of *Podospora anserina* has been sequenced^4^. SNF1 protein sequences of yeast and two filamentous fungi *Neurospora crassa* and *Hypocrea jecorina* were used as a query to identify the homolog in *P. anserina* genome. A homologous protein was found in *P. anserina*, presenting the highest identity with *N. crassa*, *H. jecorina*, and yeast, which was 75.64, 83.63, and 41%, respectively, and designated as SNF1 (Protein ID: CAP72646.1; Pa_2_770). The open reading frame (ORF) of SNF1 in *P. anserina* was predicted to be 2,127 bp in length, encoding a peptide of 708 amino acids with a predicted molecular weight of ∼79.91 kDa (Supplementary Figure S1A). SMART analysis^5^ of SNF1 suggested that this protein contained a serine/threonine (Ser/Thr) kinase catalytic domain (KD) (residues 67–318) and a two C-terminal domain (CTD) (residues 532–628 and residues 633–704). KD with the identity of 78.17% exhibited a high similarity to the SNF1 Ser/Thr catalytic domain in *Saccharomyces cerevisiae*. The degree of similarity was even lower at the CTD (25% identity), and only a few blocks showed identical residues (Supplementary Figures S1,C). Maximum likelihood method was applied to analyze the similarities among above 11 proteins, and a phylogenetic tree was constructed (Figure 1). The predicted amino acid sequence of *PaSNF1* shared an identity of 75.64% with NcSNF1 of *N. crassa* (CAD70761), 64.67% with HjSNF1 of *H. jecorina* (AF291845) (Cziferszky et al., 2003), 67.73% with FoSNF1 of *Fusarium oxysporum* (AAN32715) (Ospina-Giraldo et al., 2003), 66.06% with BbSNF1 of *Beauveria bassiana* (EJP66968) (Wang et al., 2014), 63.41% with PmSNF1 of *Pestalotiopsis microspora* (AUW40141) (Wang et al., 2018), 57.53% with MgSNF1 of *Magnaporthe grisea* (ABF48563) (Yi et al., 2008), 57.94% with SsSNF1 of *Sclerotinia sclerotiorum* (CAB40826) (Vacher et al., 2003), 44.35% with PdSNF1 of *Penicillium digitatum* (AFS18464) (Zhang et al., 2013), and 43.43% with CcSNF1 of *Cochliobolus carbonum* (AAD43341) (Tonukari et al., 2000). PaSNF1 protein showed lower homology with SNF1 protein from *S. cerevisiae*, which was 42%. SNF1 orthologs showed that PaSNF1 from different fungi clustered together, whereas yeast homologs formed a separate cluster (Figure 1).

### Creation of *PaSNF1* Knockout and Complementation Mutants

*PaSNF1* disruption mutant was generated by replacing a coding sequence of *PaSNF1* with the genetic resistance gene. The *PaSNF1* disruption mutant without additional ectopic integrations of the replacement cassette sequence mutants was confirmed by PCR and Southern blot (Supplementary Figure S2). For the complementation CP*PaSNF1*, a functional *PaSNF1* ORF was introduced into the Δ*PaSNF1* mutant, and the obtained complementation mutants were screened on a selective medium containing nourseothricin and geneticin and then confirmed by PCR.

### Effects of *PaSNF1* on Growth and Development of *Podospora anserina* in Standard Conditions

**FIGURE 2:**
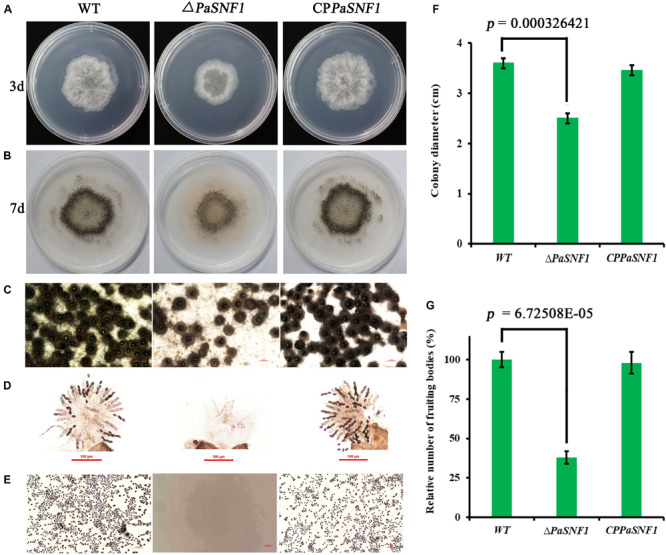
Growth and development of WT and mutants on M2 medium. **(A)** Colony size of WT and Δ*PaSNF1* after 3 days of incubation. **(B)** Fertility on M_2_ medium developed after 5 days. Perithecia are visible as small black dots. All the strains produced the usual ring of mature perithecia in the center of the Petri dish. **(C)** Microscopic observation of perithecia of WT and Δ*PaSNF1*. **(D)** Microscopic observation of ascospores inside perithecia. **(E)** Microscopic observation of ascospores erupting from fruiting bodies. **(F,G)** Colony diameter and fruiting bodies number, respectively; fruiting body number was expressed with respect to that of WT considered as 100%. Error bars are standard deviations of triplicate samples. Differences in the data were assessed by the *T*-test. WT, wild type; *PaSNF1*, *Podospora anserina* SNF1.

To investigate the effects of SNF1 in the development of *P. anserina*, the *PaSNF1* mutant (Δ*PaSNF1*), complementation mutants (CP*PaSNF1*), and the WT strains were cultured under the optimal conditions for growth and sexual reproduction (on M2 minimal medium at 27°C, in the presence of light). Then the detailed phenotypic analysis was performed. The knockout of *PaSNF1* gene led to the growth and developmental defects of *P. anserina*. Compared with that of WT, the growth of the mutant Δ*PaSNF1* was significantly decreased. Colony size of Δ*PaSNF1* was 77% of WT after 3 days of incubation (Figures 2A,F). The fruiting bodies of WT strain began to develop after 5 days. There were numerous viable ascospores expelled after 2 days (Figure 2B). Δ*PaSNF1* mat + x Δ*PaSNF1* mat- led to a defect in perithecium production and ascospore formation. Compared with WT, there was a 62% reduction in the production of perithecia (Figure 2G). Surprisingly, no ascospore was expelled from perithecia in Δ*PaSNF1* mutant (Figures 2B,E). Microscopic observation of perithecia showed that the ascus shells produced by WT and mutants were similar, without significant difference in diameter. The ascus shell of the mutant also differentiated into a normal neck (Figure 2C). Microscopic observations of squashed perithecia were made in order to observe produced ascospores and asci. Typical rosettes of asci with four ascospores were observed in WT strain crosses. Usual rosettes were composed of mature ascospores, which were dark green owing to accumulation of melanin, and not fully ripened ascospores, which were light green. However, transparent asci were observed and failed to form intact ascospores in the Δ*PaSNF1* (Figure 2D). These observations indicate that the lack of ascospore release in the Δ*PaSNF1* was due to a defect in ascospore formation rather than a defect in the mechanism of forcible ascospore discharge.

**FIGURE 3:**
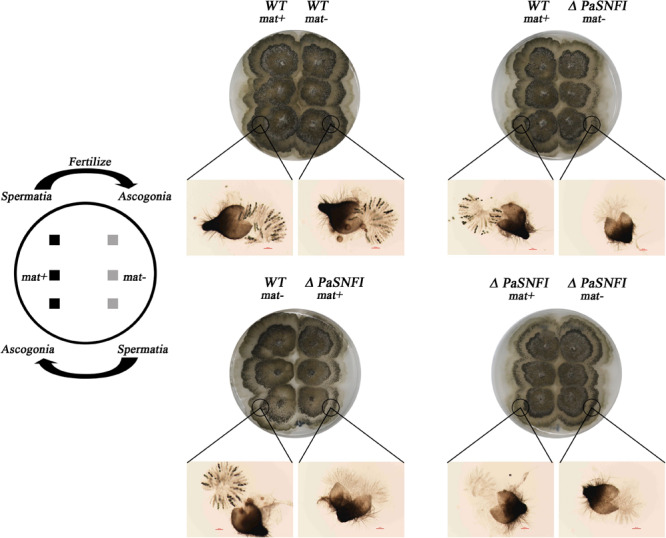
Spreading experiments. *Podospora anserina* is a heterothallic species with two mating types called mat+ and mat-. Each mating type differentiates male and female organs. Male organs (spermatia) are single cells that can be spread by water all over the plate. They are recognized by female organs of opposite mating type, which fuse with the male cells and subsequently differentiate in mature fruiting bodies. Crosses were made by inoculating the strains 1.5 cm apart. After 3 days of growth, 1.5 ml of water was added and spread all over the plate. The pictures were taken 4 days after fertilization.

To further analyze the role of the *PaSNF1* gene in sexual development, we investigated the behavior of Δ*PaSNF1* strain as male and female partners in crosses with a WT strain. Plates with Δ*PaSNF1* mat + and WT mat- mycelia were covered with 1.5 ml of sterile water, which spread male gametes from one mycelium on the other and allowed these spermatia to fertilize female organs (Figure 3). WT mat- female organs fertilized by Δ*PaSNF1* mat + spermatia produced ascospores, indicating that the deletion of *PaSNF1* is not dominant and can be complemented by the functional *PaNSF1* gene present in the WT female strain. In contrast, Δ*PaSNF1* mat + female organs fertilized by WT mat- spermatia did not produce ascospores, indicating that Δ*PaSNF1* has a maternal effect; that is, the presence of a functional *PaSNF1* gene in the male nuclei cannot complement the absence of this gene in the female organ. Crosses on plates with Δ*PaSNF1* mat- and WT mat + indicated that the maternal effect of Δ*PaSNF1* is not mating-type dependent. In addition, *SNF1* knockout resulted in a red–orange pigment deposition of the mutant Δ*PaSNF1*, compared with WT on solid M2 plates (Figure 2B). To further confirm that the phenotypic changes observed in the mutant resulted from the deletion of *PaSNF1* instead of other unknown genetic mutations, we conducted complementation analyses of Δ*PaSNF1*. The defects of Δ*PaSNF1* in mycelial growth, sexual development, and pigmentation were complemented by genetic complementation. Our results indicated that *PaSNF1* not only affected mycelial growth but also regulated fruit body formation and developmental maturation.

### The Roles of *SNF1* in the Environmental Stress Tolerance

**FIGURE 4:**
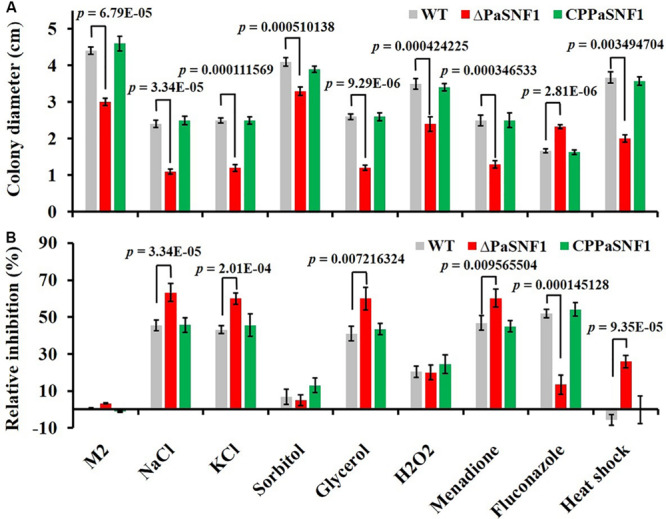
Tolerance analysis to environmental stresses. **(A)** Colony sizes of WT, Δ*PaSNF1*, and CP*PaSNF1* were incubated separately on M2 medium, supplemented with one of the osmotic stress reagents (0.5 M of NaCl, 0.5 M of KCl, 0.5 M of sorbitol, and 0.75 M of glycerol), oxidative reagents (0.01% H_2_O_2_ and 50 μM of menadione), and 2.5 μg/ml of fluconazole; and fresh mycelium was incubated at 45°C in water bath for 2 h. M2 medium without the supplements was served as the control. **(B)** Relative inhibition rate. All cultures were incubated at 27°C for 3 days, and colony size was measured. Three plates were set for each treatment, and the experiment was repeated once. Error bars are standard deviations of triplicate samples. Differences in the data were assessed by the *T*-test. WT, wild type; *PaSNF1*, *Podospora anserina* SNF1.

To determine the roles of *PaSNF1* in response to stress conditions, the growth of the strains under different stress conditions was analyzed (Figure 4). The growth of the Δ*PaSNF1* mutant was significantly inhibited by 0.5 M of NaCl, 0.5 M of KCl, and 0.75 M of glycerol, compared with that of WT and CP*PaSNF1*, whereas the result was different for sorbitol. This result suggested that *PaSNF1* may be necessary for the cellular responses of *P. anserina* to osmotic pressure. In contrast, the Δ*PaSNF1* mutant was insensitive to 0.01% H_2_O_2_. However, on plates supplemented with 50 μM of oxidant menadione, the growth rate of Δ*PaSNF1* mutant was obviously slower, implying the roles of *SNF1* in its tolerance to this stress. The Δ*PaSNF1* mutant showed similar sensitivity to heat shock, presented with slower growth, and significantly increased inhibition rate. Particularly interestingly, the resistance of the Δ*PaSNF1* mutant to the fungicide fluconazole was significantly increased after knocking out of the *SNF1* gene; compared with WT and CP*PaSNF1* strains, the growth of Δ*PaSNF1* mutant was significantly increased while the inhibition rate was reduced. These results indicated that the *PaSNF1* gene in *P. anserina* played an important role in its response to osmotic stress, oxidative stress, and heat shock.

### The Regulation of *SNF1* for Sterigmatocystin Biosynthesis

**FIGURE 5:**
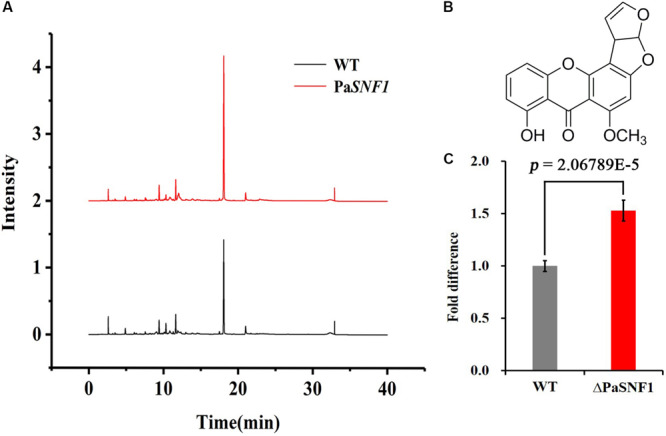
Identification and analysis of ST. **(A)** HPLC analysis of SM production with WT and Δ*PaSNF1* mutant. The strains were grown on M_2_ media for 7 days. **(B)** The chemical structure of ST by NMR. **(C)** Relative amounts of ST in Δ*PaSNF1* strain compared with WT. Error bars are standard deviations of triplicate samples. Differences in the data were assessed by the *T*-test. ST, sterigmatocystin; HPLC, high-performance liquid chromatography; SM, secondary metabolite; WT, wild type; *PaSNF1*, *Podospora anserina* SNF1; NMR, nuclear magnetic resonance.

In fungi, SMs were generally associated with the developmental processes in response to various abiotic or biotic external stimuli (Yin et al., 2012). To examine the regulation effects of *SNF1* in biosynthesis of SMs in *P. anserina*, the profile of secondary compounds in Δ*PaSNF1* cultures was analyzed by HPLC. We found that the profile of SMs was significantly altered after the deletion of *PaSNF1* (Figure 5A). Based on HPLC profiling, peak of SM 1 (at 18.5 min) in Δ*PaSNF1* was 50% higher than that of WT (Figures 5A,C). To further identify the compound at 18.5 min, large-scale fermentation was performed; the metabolites were further isolated and purified. The compound was identified as ST by NMR (Figure 5B and Supplementary Figures S3, S4). The result suggested that the biosynthesis of ST was negatively regulated by *PaSNF1*. The above results demonstrated that *PaSNF1* was a regulator of SMs and may be involved in either activation or silencing of certain gene clusters in *P. anserina*.

### Knockout of *SNF1* Led to Impaired Cellulose-Utilizing Ability

**FIGURE 6:**
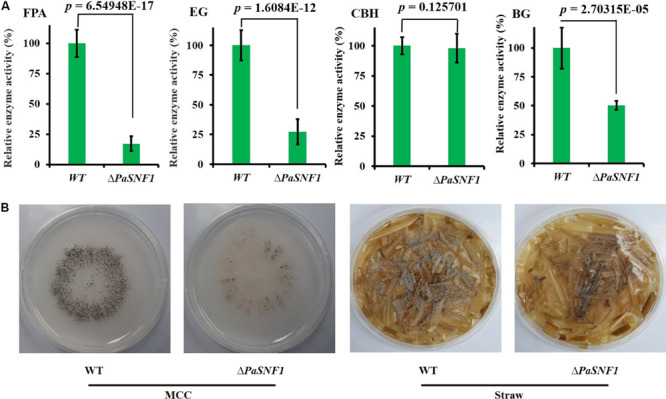
Knockdown of *PaSNF1* led to defects in cellulose-utilizing ability. **(A)** Enzyme assays in WT and Δ*PaSNF1* mutant. The mycelia of 2-day-old cultures grown on M2 medium of the indicated strains were harvested and smashed in proper extraction buffer. Activity was expressed with respect to that of WT considered as 100%. Error bars are standard deviations of triplicate samples. Differences in the data were assessed by the *T*-test. **(B)** Fertility of WT and Δ*PaSNF1* on medium with MCC and straw medium. Fertility was measured by counting the number of mature perithecia on plates containing MCC and straw medium as sole carbon source. Pictures of mycelia of WT and Δ*PaSNF1* mutant were taken after 7 days of growth. Fruiting bodies (perithecia) are visible as small black dots. *PaSNF1*, *Podospora anserina* SNF1; WT, wild type; MCC, microcrystalline cellulose.

To investigate whether the PaSNF1 kinase participated in the production of cellulase, the cellulase activity and cellulose-utilizing ability of WT and Δ*PaSNF1* were detected. The cellulase activity of WT and Δ*PaSNF1* was further compared, including FPA, EG, CBH, and BG (Figure 6A). After 3 days of culture on M2 medium, the activities of FPA, EG, and BG were significantly lower than those of WT, which were reduced to 17.2, 27.4, and 50.3%, respectively, of WT (Figure 6A). After 7 days of culture on MCC agar medium (with microcrystalline cellulose as carbon source instead of dextrin), the fruit body formation of Δ*PaSNF1* mutant was significantly reduced, and similar phenotypes were observed on straw medium (Figure 6B). Therefore, *PaSNF1* was considered as critical for the cellulose-utilizing ability of *P. anserina*.

### *SNF1* Regulated the Expression of the Carbohydrate-Active Enzymes Genes and Transporters in *Podospora anserina*

**FIGURE 7:**
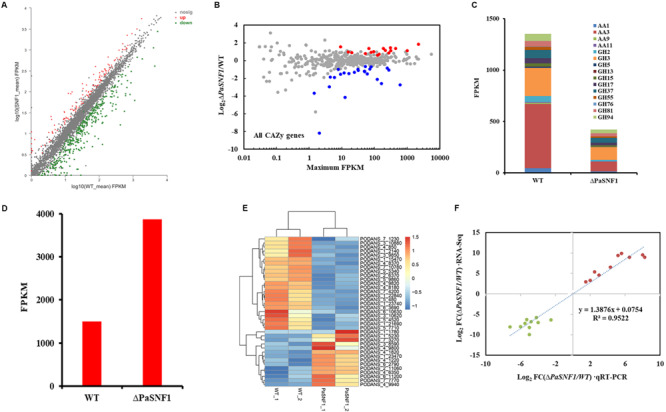
Comparative transcriptomics analysis between Δ*PaSNF1* and WT grown in Avicel medium. **(A)** Differentially expressed genes in the mutant Δ*PaSNF1* relative to WT. The expression profile was calculated for conditions including Avicel. Genes that showed differential expression identified by DESeq2 package are shown. Up-regulated and down-regulated genes are shown in red and green, respectively. **(B)** Relative mRNA abundance of all CAZy genes in Δ*PaSNF1* mutant versus WT. The significantly up-regulated genes are shown in red, whereas down-regulated genes are shown in blue (based on adjusted *p*-value). **(C)** Total expression of genes encoding major cellulases from RNA-Seq data in WT and Δ*PaSNF1* strains. **(D)** Total expression of genes encoding LPMO from RNA-Seq data in WT and Δ*PaSNF1* strains. **(E)** Regulation of putative transporter genes by *PaSNF1*. Values in heatmaps were calculated by log2 (Gene_FPKM in Δ*PaSNF1*/Gene_FPKM in WT). FPKM mapped. **(F)** Expression level validation of DEGs using RT-qPCR. Comparisons of log2 ratios of 10 up-regulated and 10 down-regulated genes from the RNA-Seq data and RT-qPCR experiments with paired primers (Supplementary Table S1). *PaSNF1*, *Podospora anserina* SNF1; WT, wild type; CAZy, Carbohydrate-Active Enzymes; DEG, differentially expressed genes.

The results described above confirmed that *SNF1* made effects on cellulase and hemicellulase expression of *P. anserina*; however, these effects could not be validated solely from growth data. To determine whether the defect in cellulase secretion and activity in the Δ*PaSNF1* mutant was due to the failure to induce cellulase gene expression versus a defect in cellulase secretion, we assessed genome-wide expression differences via RNA-Seq between the WT and Δ*PaSNF1* strains in medium containing Avicel. Transcriptome results showed that significant expression differences in 468 genes were observed in Δ*PaSNF1* compared with WT; DEGs accounted for 4.3% of all genetic data, among which 129 genes were up-regulated and 339 genes were down-regulated (Figure 7A and Supplementary Table S2). Among these genes, 41 of 471 CAZyme encoding genes were detected (Carbohydrate Active Enzymes database^6^), 24 genes were significantly down-regulated, 17 genes were significantly up-regulated (Figure 7B and Supplementary Table S3), and the DEGs accounted for 8.7% of all CAZyme-encoding genes. To verify whether the CAZyme-encoding genes were enriched and overexpressed, we used Log2FC(Δ*PaSNF1*/WT) to perform statistical analysis (*T*-test) on up-regulated and down-regulated genes between CAZyme-encoding genes and non-CAZyme-encoding genes in DEGs. The results indicate that the differences of up-regulated (*p* value = 0.219811) and down-regulated genes (*p*-value = 0.740564) between CAZyme-encoding genes and non-CAZyme-encoding genes were not significant. However, the proportion (41/468 × 100% = 8.76%) of CAZyme-encoding genes in DEGs was doubled as compared with the proportion (471/10,888 × 100% = 4.33%) of all CAZyme-encoding genes in the genome, and these results suggest to some extent that more CAZyme-encoding genes were induced to be activated, although CAZyme-encoding genes are not overrepresented in the DEGs in Δ*PaSNF1*. These down-regulated genes encoded CAZymes in the glycoside hydrolase family (GH2, GH3, GH5, GH13, GH17, GH37, GH55, GH76, GH81, and GH94), laccase (AA1 and Lac), cellobiose dehydrogenases (AA3 and CDH), and lytic polysaccharide monooxygenases (LPMO, AA9, and AA11); however, in the Δ*PaSNF1* mutant, FPKM of LPMOs was very small, and the FPKM of AA9 and AA11 is 0.665 and 3.83, respectively (Figure 7C and Supplementary Table S3). The majority of which comprised glycosyl hydrolases (GHs) participated in cellulose and hemicellulose degradation. These findings implied that expression of most of the cellulolytic and hemicellulolytic genes was affected by the deletion of *PaSNF1*. Down-regulated genes also contained some glycosidase activity against various side chains in hemicellulose, such as endo-1,6-alpha-mannosidase (PODANS_1_19460), β-galactosidase (PODANS_1_18330), chitin deacetylase (PODANS_1_4780), and α,α-trehalase (PODANS_1_2050). The reduced expression of various glycosidase genes was also reflected in the decreased ability to grow on their oligosaccharide substrates (Supplementary Figure S5). The majority of up-regulated genes encoded CAZymes are the glycoside hydrolase family (GH6, GH7, GH11, GH18, and GH53) and LPMOs (AA9: PODANS_1_21900, PODANS_2_4860, PODANS_3_2580, PODANS_5_10760, PODANS_6_11370, and PODANS_6_11470). FPKM of LPMOs was significantly increased, which reached 69% in up-regulated genes encoded CAZymes in Δ*PaSNF1*, 2.5 times that of WT (Figure 7D), which may be related to the increase of H_2_O_2_ in the mutant. In addition, 23 transporters were found to be down-regulated in the Δ*PaSNF1* strain compared with WT, and nine of these genes encoded proteins that belong to the major facilitator superfamily (MFS) permeases (Figure 7E and Supplementary Table S4). Four sugar transporters were included: *PODANS_5_4520* (*SPT1*) encoded sugar and polyol transporter 1 (SPT1) (Schilling and Oesterhelt, 2007), *PODANS_4_9820* (*Hgt-1*) was identified as a high-affinity glucose transporter (Baruffini et al., 2006), and *PODANS_5_480* and *PODANS_1_22840* (*MstA*) showed the capability of transporting monosaccharides including xylose (Jorgensen et al., 2007). However, none of the transcription factors known to directly regulate cellulase gene expression exhibited altered expression. Interestingly, two genes (*PODANS_3_3440* and *PODANS_5_12360*) encoding secondary metabolic regulators LAE1 were significantly down-regulated (Supplementary Table S2), which was a homolog of methyltransferase LAE1 of *Trichoderma reesei*. It was involved in cellulase gene expression regulations (Seiboth et al., 2012), suggesting an indirect relationship between SNF1 and cellulase expression. To validate the DEGs identified from RNA-Seq data, transcript levels of 10 up-regulated (*PODANS_1_9380*, *PODANS_1_5230*, *PODANS_1_12700*, *PODANS_4_5380*, *PODANS_5_9670*, *PODANS_6_2530*, *PODANS_7_8775*, *PODANS_4_5370*, *PODANS_2_7185*, and *PODANS_1_990*) and 10 down-regulated genes (*PODANS_3_1840*, *PODANS_6_9965*, *PODANS_7_150*, *PODANS_5_3595*, *PODANS_3_2688*, *PODANS_3_780*, *PODANS_6_10085*, *PODANS_5_11880*, *PODANS_1_19460*, and *PODANS_7_8100*) in the mycelium cells of Δ*PaSNF1* and WT were assessed via RT-qPCR with paired primers (Supplementary Table S1) and compared with those in the RNA-Seq data. As a result, all the gene transcripts assessed in RT-qPCR showed the same up-regulated or down-regulated trends as determined by the RNA-Seq analysis for DEGs identification (Figure 7F).

## Discussion

Organisms would suffer a variety of nutritional conditions during their life cycle. Complex signaling networks have been evolved in microbes to identify and prioritize the utilization of available energy sources, such as the CCR system. The CCR system would prevent the utilization of difficultly metabolizable carbon sources for ensuring the preferential utilization of easily metabolizable carbon sources such as *D*-glucose. For many fungi, several genes involved in the carbon catabolism were regulated by CCR, including the genes encoding carbohydrate-degrading enzymes such as cellulase, amylase, and xylanase. The SNF1/AMPK was highly conserved among eukaryotic organisms, which was a key regulator in both carbon metabolism and energy homeostasis. The regulation of SNF1 activity played a central role in the alleviation of CCR (Brown et al., 2013). During the absence of glucose, *SNF1* regulated the expression of metabolic genes by controlling transcriptional activators and repressors (Carlson, 1999). In *Saccharomyces cerevisiae*, *SNF1* acted as an activator for more than 400 glucose-repressing genes, some of which were involved in alternative carbon source utilization pathways (Young et al., 2003). In this study, the function of *PaSNF1* was explored. Our results demonstrated that *PaSNF1* played important roles in regulating vegetative growth, sexual development, stress responses, and biosynthesis of SMs in *Podospora anserina* and then confirmed that it was a major actor of lignocellulose degradation in *P. anserina*.

The PaSNF1 protein consisted of a serine/threonine (Ser/Thr) kinase catalytic domain (KD) (residues 67–318) and a two CTD (residues 532–628 and 633–704), showing the same structure with the SNF1s identified in many other organisms (Zhang et al., 2013; Islam et al., 2017). In addition, the PaSNF1 protein generally shared a high sequence identity with the SNF1s of other organisms, especially the catalytic domain (Cziferszky et al., 2003; Ospina-Giraldo et al., 2003; Wang et al., 2014, 2018). Phylogenetic analysis of SNF1 homologous proteins from filamentous fungi and yeasts formed a separate cluster. Results of phylogenetic analysis indicated that PaSNF1 was much more closely related to the SNF1 orthologs from the filamentous ascomycetous fungi than those from ascomycetous yeasts, plants, or mammals (Figure 1). Among the SNF1s in the filamentous fungi, the PaSNF1 was closest to the SNF1 ortholog identified in *Neurospora crassa*, *Hypocrea jecorina*, *Fusarium oxysporum*, and *Beauveria bassiana* (Figure 1).

The essential role of *SNF1* in filamentous fungi was the regulation of vegetative growth and sexual development. The ability to form the perithecia and ascospores was affected in *GzSNF1* deletion mutants (Lee et al., 2009), conidiation was also affected by the loss of *PdSNF* (Zhang et al., 2013), and the ablation of *LmSNF1* would result in impaired sporulation and spore germination (Feng et al., 2014). In Δ*MoSNF1* mutant, the sporulation ability was abolished (Yi et al., 2008), and the *PmSNF1* also played a critical role in conidiation and vegetative growth in *Pestalotiopsis microspora* NK17 (Wang et al., 2018). Deletion of *SNF1* in *Fusarium graminearum* led to defects in vegetative growth and sexual reproduction (Yu et al., 2014). Our observations were consistent with the above results; the knockout of *PaSNF1* gene led to the growth and developmental defects of *P. anserina*. The *PaSNF1* did not only affect mycelial growth but also regulate fruit body formation and developmental maturation (Figure 2). This finding suggested that *PaSNF1* could trigger the expression of genes required for the formation of fruit body and ascospores. Mechanisms underlying these functions may be associated with the inadequate or unbalanced nutrients (Lee et al., 2009).

In yeast, the SNF1 protein kinase has been necessary for its response to various environmental stresses such as sodium and lithium salts, alkaline pH, heat shock, and hyperosmolarity (Hong and Carlson, 2007). Treatment with high concentrations of salts (0.8 M of KCl and 1 M of NaCl), 1 M of sorbitol, and 2 mM of oxidant H_2_O_2_ could dramatically inhibit vegetative growth of the Δ*PmSNF1* mutant strain (Wang et al., 2018). However, the response of the *SNF1* mutant of *Cryptococcus neoformans* to osmotic, salt, and oxidative stresses was not significantly different from that of the WT (Hu et al., 2008). Our results showed that the growth of the Δ*PaSNF1* mutant would be significantly inhibited by 0.5 M of NaCl, 0.5 M of KCl, and 0.75 M of glycerol; however, Δ*PaSNF1* mutant was not sensitive to 0.5 M of sorbitol. A comparable function for *SNF1* in the *C. neoformans* JEC21 has been reported (Yang et al., 2010). Our data clearly demonstrated the crucial roles of *PaSNF1* in the tolerance of fungus to heat shock; the mycelium of the Δ*PaSNF1* mutant was sensitive to heat shock of 45°C. This function of SNF1 has been previously reported in other fungi, such as *C. neoformans* and *Pestalotiopsis microspora* (Yang et al., 2010; Wang et al., 2018). Moreover, after the knockout of *SNF1* gene, the resistance of the Δ*PaSNF1* mutant to the fungicide fluconazole would be significantly increased, whereas the sensitivity to fluconazole was not varied in Δ*CnSNF1* mutant (Hu et al., 2008). It indicated the remarkable functional deviation between *PaSNF1* and *CnSNF1*, although the underlying mechanism remained to be identified.

Ascomycete genomes code for on average 40 biosynthetic gene clusters with crucial importance in SM synthesis (Pusztahelyi et al., 2015). Genome analyses in aspergilli suggest between 39 and 80 biosynthetic gene clusters per species (Inglis et al., 2013). Sequence analysis of the *P. anserina* genome revealed its ability to produce 40 putative SMs including 18 polyketide synthases (PKSs) (Espagne et al., 2008). However, until now, only limited chemical investigations have been conducted, and plenty of SMs were yet to be discovered. Recent studies have shown that ABR1 was involved in biosynthesis of DHN melanin, which colored ascospores; and knockout of ABR1 led to decreased ascospore pigmentation accompanied by lowered ascospore germination rate (Xie et al., 2018). Shen et al. (2019) reported that *PaStcA* and *PaAflR* were key genes necessary for the biosynthesis of ST in *P. anserina*, deletion of *PaStcA* that blocked the ST pathway, and overexpression of *PaAflR* that resulted in the excessive accumulation of ST, thus leading to a female sterility phenotype. In this study, to identify the metabolites regulated by *PaSNF1*, the large-scale fermentation of the Δ*PaSNF1* mutant was performed, and the structures of metabolites with unique peaks were characterized. This result suggested that *PaSNF1* may have negative effects on the biosynthesis of ST. The general pattern of the peaks for ST was significantly increased in the HPLC profile of Δ*PaSNF1* mutant compared with WT (Figure 5). Correspondingly, the deletion of *PaSNF1* led to a defect in perithecium production and ascospore formation. Moreover, *OE-PaAflR* displayed overproduction of ST, and overexpression of *PaAflR* also led to sterility (Shen et al., 2019). These results indicated that ST was closely related to the development of fungi and spore formation. To our knowledge, this has been the first report of SNF’s regulation effects on ST biosynthesis.

In addition to regulating ST synthesis, SNF1 also played a critical role in regulating cellulose degradation. We showed that a Δ*PaSNF1* mutant displayed severe growth defects on cellulose, and similar phenotypes were observed on straw medium (Figure 6B), which was correlated with a lack of cellulolytic enzyme activity. Indeed, the enzyme activities of FPA, EG, and BG in Δ*PaSNF1* mutant were significantly reduced (Figure 6A). The defects in *PaSNF1* would lead to the reduced (or impaired) production of such enzymes, thus inhibiting the degradation of lignocellulose and utilization of carbon sources. Consistent with this phenotype, the expression of the many CAZymes genes of *P. anserina* was significantly decreased in the Δ*PaSNF1* mutant compared with WT (Figure 7). Following the extracellular degradation of the lignocellulose, the uptake of the soluble breakdown products has been a key process to regulate the transcription of cellulases and related genes. The transporters played an important role, some of which exhibited the ability to sense the breakdown products during their passage through the cell membrane (Novy et al., 2019). Transcriptome analysis showed that the genes coding for MFS permeases were also highly repressed in the Δ*PaSNF1* mutant, including four putative sugar transporters. These findings suggested that *PaSNF1* played an important role in positively regulating the expression of genes encoding cellulolytic enzymes. Interestingly, our data revealed that the main transcription factors that regulated the expression of cellulases in *P. anserina* showed no change of expression in the mutant strain relative to WT strain. For example, the expression of XYR1, CLR1, and CLR2 was not significantly varied in mutant strains, especially CRE1, which was the key protein of CCR and the direct target protein of SNF1. The expression of cellulosic biomass degrading enzyme genes was regulated by various global regulators. The protein methyltransferase LAE1 was one global regulator of SM gene clusters such as ST formation, which regulated sexual and asexual developmental processes in Ascomycota (Tani et al., 2014). LAE1 also played an important role in regulating the expression of cellulase, polysaccharide hydrolases, and xyr1 in *Trichoderma reesei* (Seiboth et al., 2012). Our results showed that two putative LAE1 (*PODANS_3_3440; PODANS_5_12360*) expressions were significantly down-regulated in the Δ*PaSNF1*. This result suggested a refined dual mechanism that controlled the expression of cellulase genes through the modulation of transcription factors.

SNF1 promoted feeding on lignocellulose and reduced the synthesis of ST. There may be a balance between feeding and fighting competitors on the basis of anticorrelated effects on each pathway. In our study, negative correlation was observed based on the data of enzyme activity and metabolite map. In a previous study, analysis of the genome revealed that CAZymes of *T. reesei* were often observed in clusters along with genes involved in SM (Martinez et al., 2008). It was considered as a means to fend off competitors for nutrients. Fungi constantly faced the challenge to outcompete other organisms in complex ecosystems. Therefore, they developed powerful enzyme systems for degradation of substrates, which provided for fast growth and efficient colonization of their environment (van den Brink and de Vries, 2011). However, fungi also evolved the ability to kill, or at least inhibit the growth of their competitors, by producing a versatile array of SMs (Demain and Fang, 2000). The application of these different survival utilities should be tightly controlled in order to balance the assignment of resources for feeding to succeed by superior growth or fighting to decrease the chances of survival for competitors. This study supported this hypothesis of coordination of substrate degradation and competition. This may be a mechanism via which fungi evolved to balance the operation of primary and secondary metabolism during its life cycle. Thus, an economic distribution of resources for feeding (enzyme production) and fighting (SM production) would be reasonable.

## Conclusion

In conclusion, in this work, the roles of *PaSNF1* in vegetative growth, sexual development, and lignocellulose degradation in *Podospora anserina* were investigated. Our study clearly suggested that SNF1 played a critical regulation role in growth, development, and stress response. Importantly, SNF1 can function as a critical global regulator for the SM biosynthesis and cellulase production. In the future, proteomics analysis and ChIP-seq on PaSNF1 would be performed to identify the interaction partners of PaSNF1. This will be crucial to develop deeper understanding on the cellulase-regulating signal transduction processes and the SNF1-mediated regulation mechanism of lignocellulose degradation in *P. anserina*, as well as its effects on growth and development.

## Data Availability Statement

The datasets generated for this study can be found in the SRA database: https://www.ncbi.nlm.nih.gov/sra/PRJNA597840, accession number PRJNA597840.

## Author Contributions

YL, PY, and NX designed the study. YL, XL, YQ, and SL performed the experiments. YL, GL, SL, and LM analyzed the data. YL and NX wrote the manuscript. All authors reviewed the results and approved the final version of the manuscript.

## Conflict of Interest

The authors declare that the research was conducted in the absence of any commercial or financial relationships that could be construed as a potential conflict of interest.

## Footnotes

1 http://podospora.i2bc.paris-saclay.fr/

2 https://www.ncbi.nlm.nih.gov/genome/?term=txid5145[orgn],

3 http://www.r-project.org

4 http://podospora.i2bc.paris-saclay.fr/index.php

5 http://smart.embl-heidelberg.de/

6 http://www.cazy.org/
