# Supplementary material for: Involvement of PaSNF1 in Fungal Development, Sterigmatocystin Biosynthesis, and Lignocellulosic Degradation in the Filamentous Fungus Podospora anserina
Source: Front Microbiol. 2020 Jun 10;11:1038. doi: 10.3389/fmicb.2020.01038 (PMC7299030; doi:10.3389/fmicb.2020.01038)
Supplement: Supplementary file 5 [file Data_Sheet_1.docx]

Supporting Information for

**Functional characterization of *PaSNF1* in the filamentous fungus *Podospora anserina*: roles in fungal development, stress responses, sterigmatocystin biosynthesis and lignocellulosic degradation**

**Yuanjing Li ^1^, Pengfei Yan ^2^, Xiaojie Lu^1^, Shang Liang^1^, Gang Liu^1^, Shuangfei Li^1^, Ning Xie^1^***

^1.^ Shenzhen Key Laboratory of Microbial Genetic Engineering, College of Life Sciences and Oceanography, Shenzhen University, Shenzhen 518060, P. R. China;

^2.^ Key Laboratory of Functional Inorganic Material Chemistry (MOE), School of Chemistry and Materials Science, Heilongjiang University, Harbin 150080, P. R. China;

*.  Co-corresponding author

*. To whom correspondence should be addressed: Ning Xie, Shenzhen Key Laboratory of Microbial Genetic Engineering, College of Life Sciences and Oceanography, Shenzhen University, Shenzhen 518060, P. R. China. E-mail: ning.xie@szu.edu.cn.

**Supplementary Figure Legends**

**Fig. S1. Amino acid sequence alignment and bioinformatics analysis.** (A)Schematic diagram of the structure of SNF1 protein from *P. anserina* and SNF1 homologs from *S.cerevisiae*. This protein contained a catalytic kinase domain (KD) and a two C-terminal domain (CTD), the overall percentage of amino acid sequence identity with ScSNF1 is shown on the right. (B) and (C) Amino acid sequence alignment of KD (78.17 % identity) and CTD (25 % identity) KD and CTD with SNF1 homologs in *S. cerevisiae*. The identical amino acids were starred and highlighted in blue and the less conserved amino acids were shown in turquoise.

**Fig. S2. Construction of ∆*PaSNF1* mutant and production of mutant cultures protoplast transformation.** (A) Upstream and downstream of the *PaSNF1* gene was amplified from the genomic DNA of *P. anserina* using specific primers (*PaSNF1A*, *PaSNF1B*, *PaSNF1C*, and *PaSNF1D*). The geneticin resistant gene fragment was amplified from pBC- Geneticin vector plasmid, gene knockout expression boxes *SNF1-G* and *G-SNF1* were constructed by fusion PCR. The Gene knockout expression boxes *SNF1-G* and *G-SNF1* were then introduced into the wild-type *P. anserina* through transformation (see the “Materials and methods” section). The fragment A, B line shown in the figure indicates the sequence region used in PCR verification and Southern blot analysis. (B) The results of PCR verification of the deletion in the mutants. Two fragments, 1.5 kb and 1.4 kb, were obtained by PCR amplification for ∆*PaSNF1*, while no band was seen for *P. anserina*. (C) Restriction endonuclease digestion diagram of the wild type and mutant, the wild type was cut into two pieces by Hind III (6330 bp and 5403 bp), the fragment length in the mutant was 11642 bp. (D) Southern blot for confirming the deletion of ∆*PaSNF1*. Genomic DNAs from wild type and mutant transformants were digested with Hind III. Two fragment sizes from the wild-type *P. anserina* are 5.4 and 6.3 kb, while in mutant (∆*PaSNF1*), one band of 11.6 kb was observed.

**Fig. S3. 13C-NMR of ST.** NMR spectra were performed on a Bruker Avance III 500 spectrometer (600 MHz). DMSO-d6 was applied as the solvent for analyzing.

**Fig. S4. 1H-NMR of ST.** NMR spectra were performed on a Bruker Avance III 500 spectrometer (600 MHz). DMSO-d6 was applied as the solvent for analyzing.

**Fig. S5 Growth of WT and Δ*PaSNF1* mutants on medium with different carbon sources.** WT, Δ*PaSNF1* and CP*PaSNF1* were incubated separately on medium with different carbon sources, which was M_2_ medium deleted 0.5 % dextrin then supplemented with 0.5 % Xylan, 0.5 % Mannose, 0.5 % Maltose, 0.5 % Starch and 0.5 % α-lactose, respectively. The plates were then incubated at 27 °C for two days.

**Supplementary Tables**

**Table S1. Primers used in this study**

**Table S2: Differentially expressed genes in Δ*PaSNF1* versus WT on Avicel.**

**Table S2: Differentially expressed genes of Cazys enzymes in Δ*PaSNF1* versus WT on Avicel.**

**Table S4: Differentially expressed genes of transporters in Δ*PaSNF1* versus WT on Avicel.**
