# Supplementary figures and images for: Involvement of PaSNF1 in Fungal Development, Sterigmatocystin Biosynthesis, and Lignocellulosic Degradation in the Filamentous Fungus Podospora anserina
Source: Front Microbiol. 2020 Jun 10;11:1038. doi: 10.3389/fmicb.2020.01038 (PMC7299030; doi:10.3389/fmicb.2020.01038)

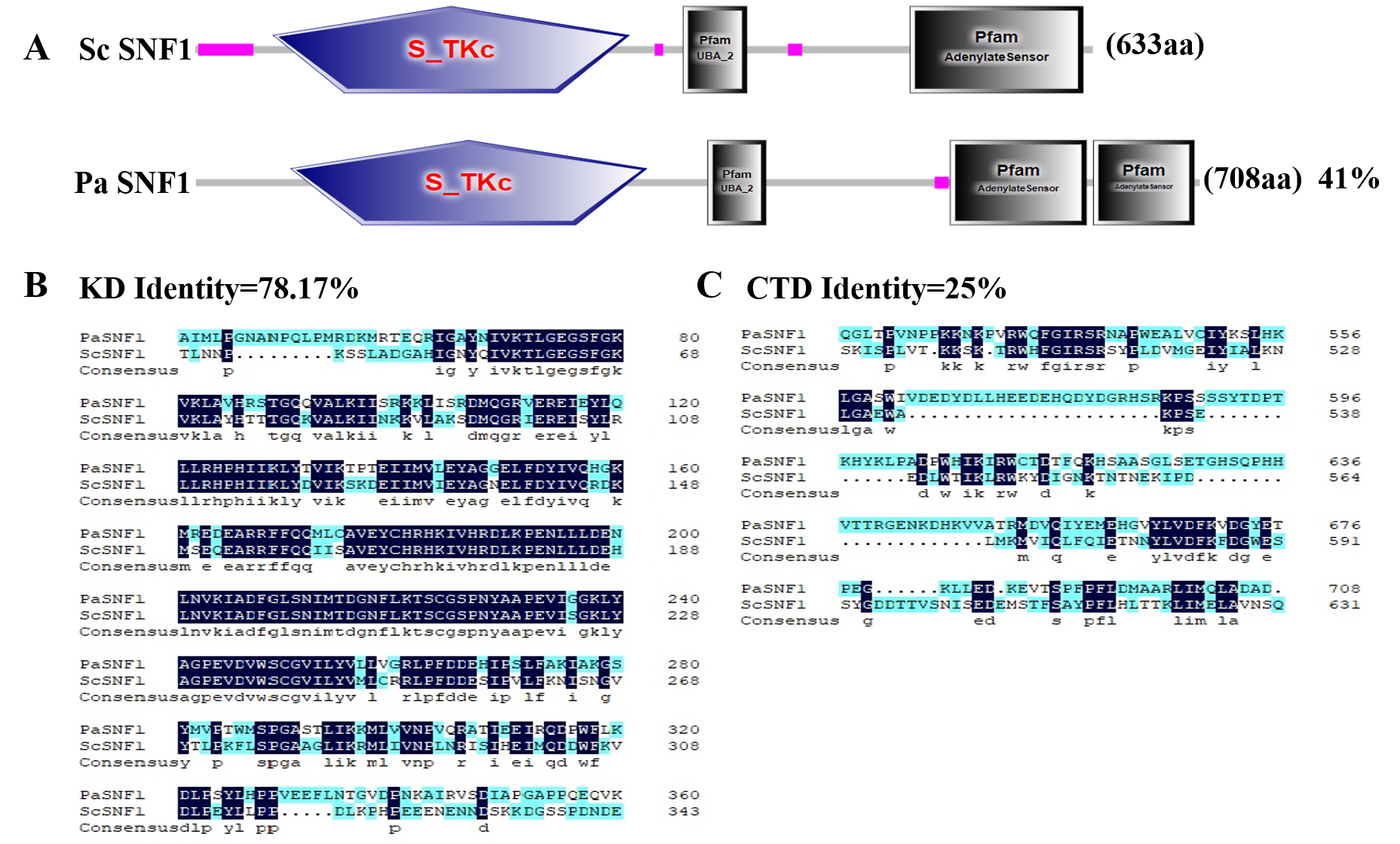

Supplement: Supplementary file 6 [file Image_1.png]

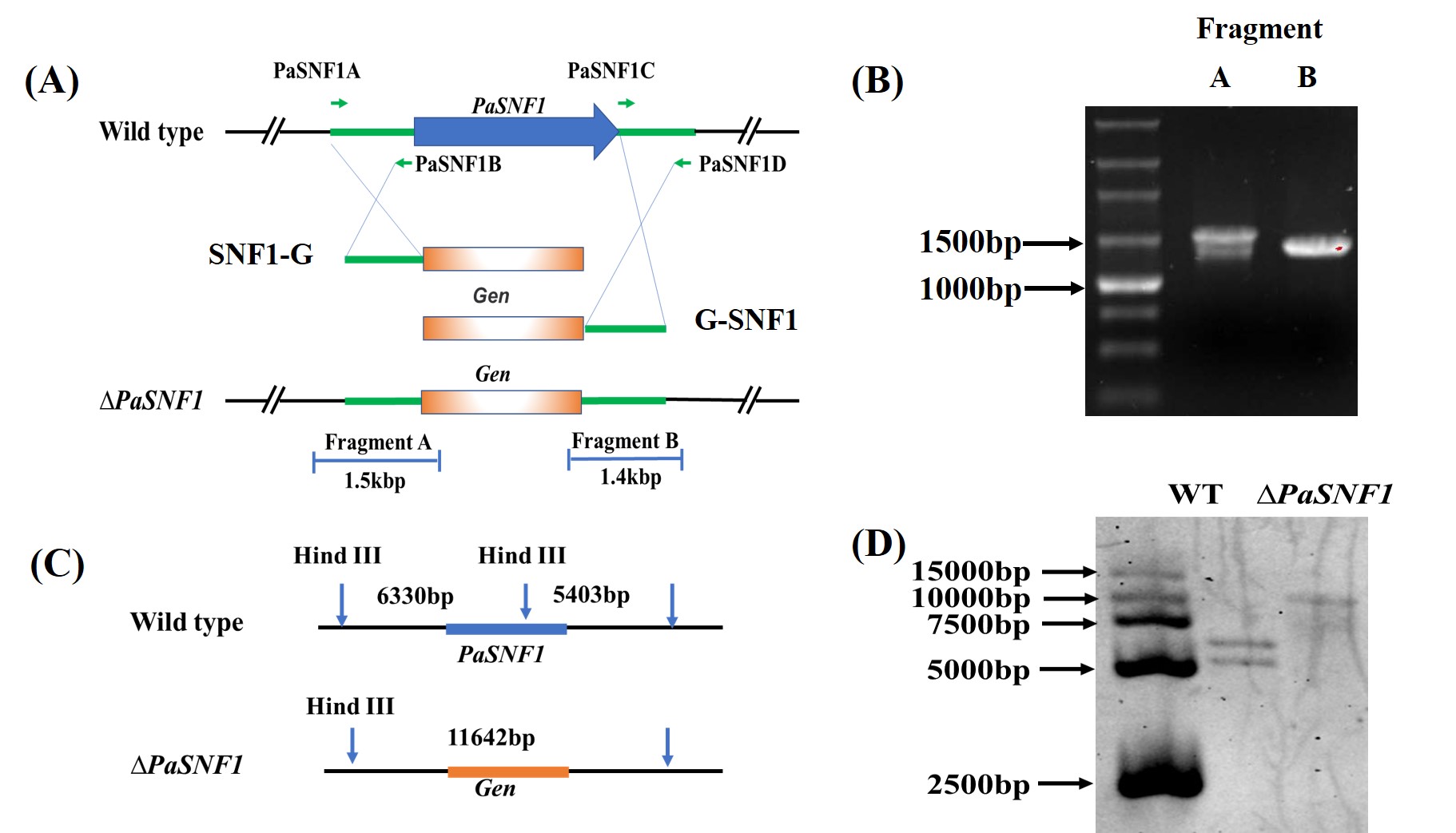

Supplement: Supplementary file 7 [file Image_2.jpg]

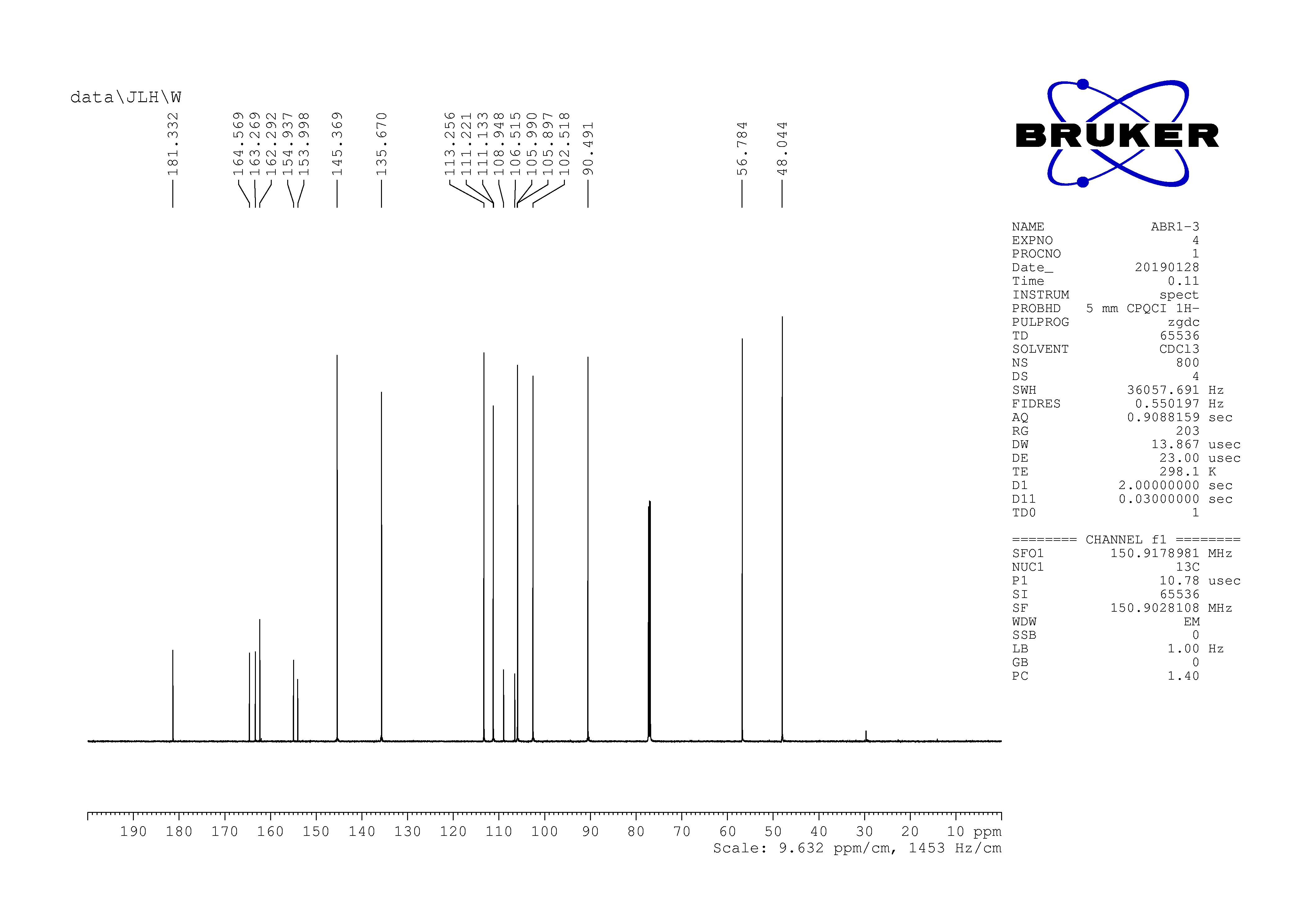

Supplement: Supplementary file 8 [file Image_3.jpg]

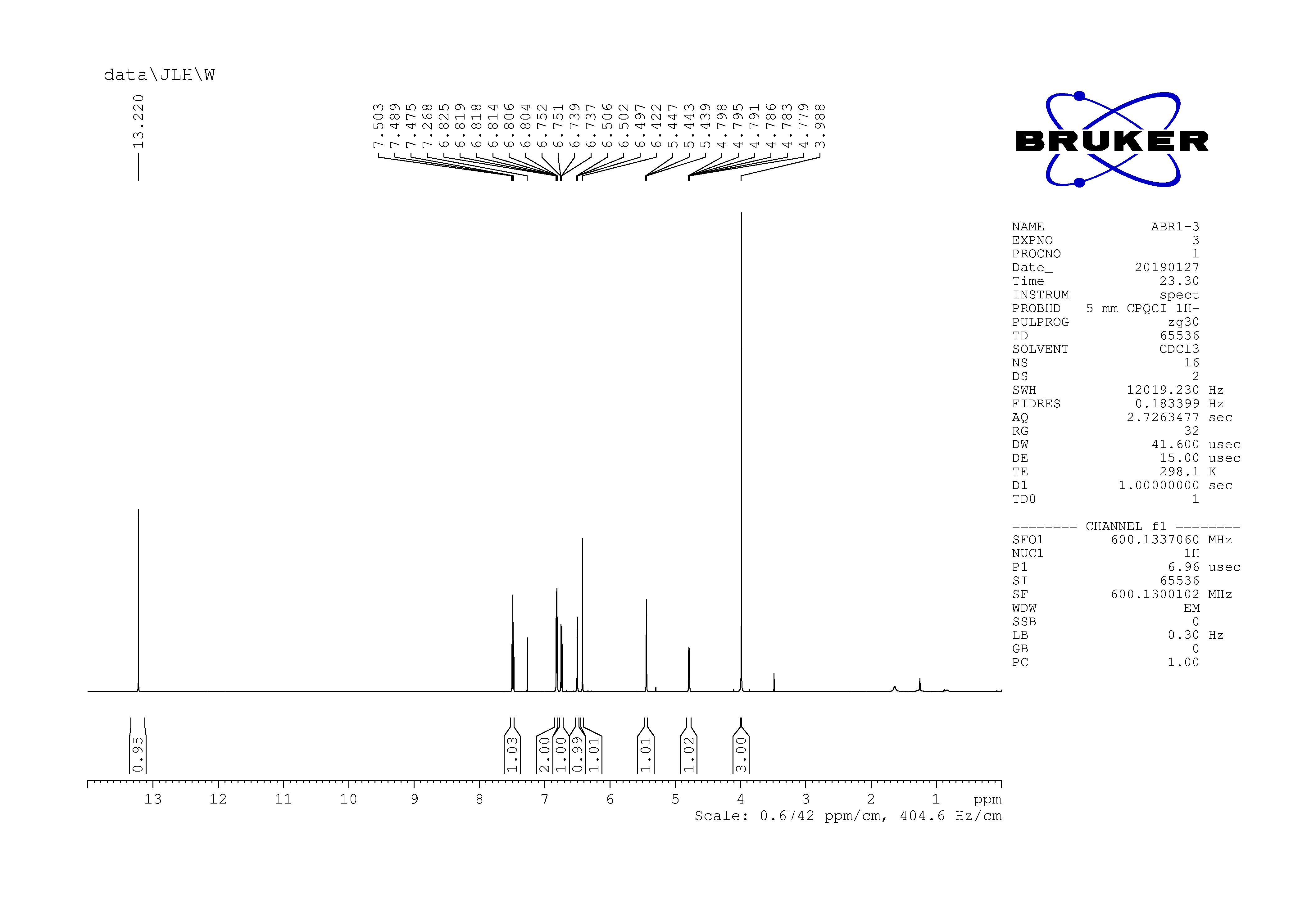

Supplement: Supplementary file 9 [file Image_4.jpg]

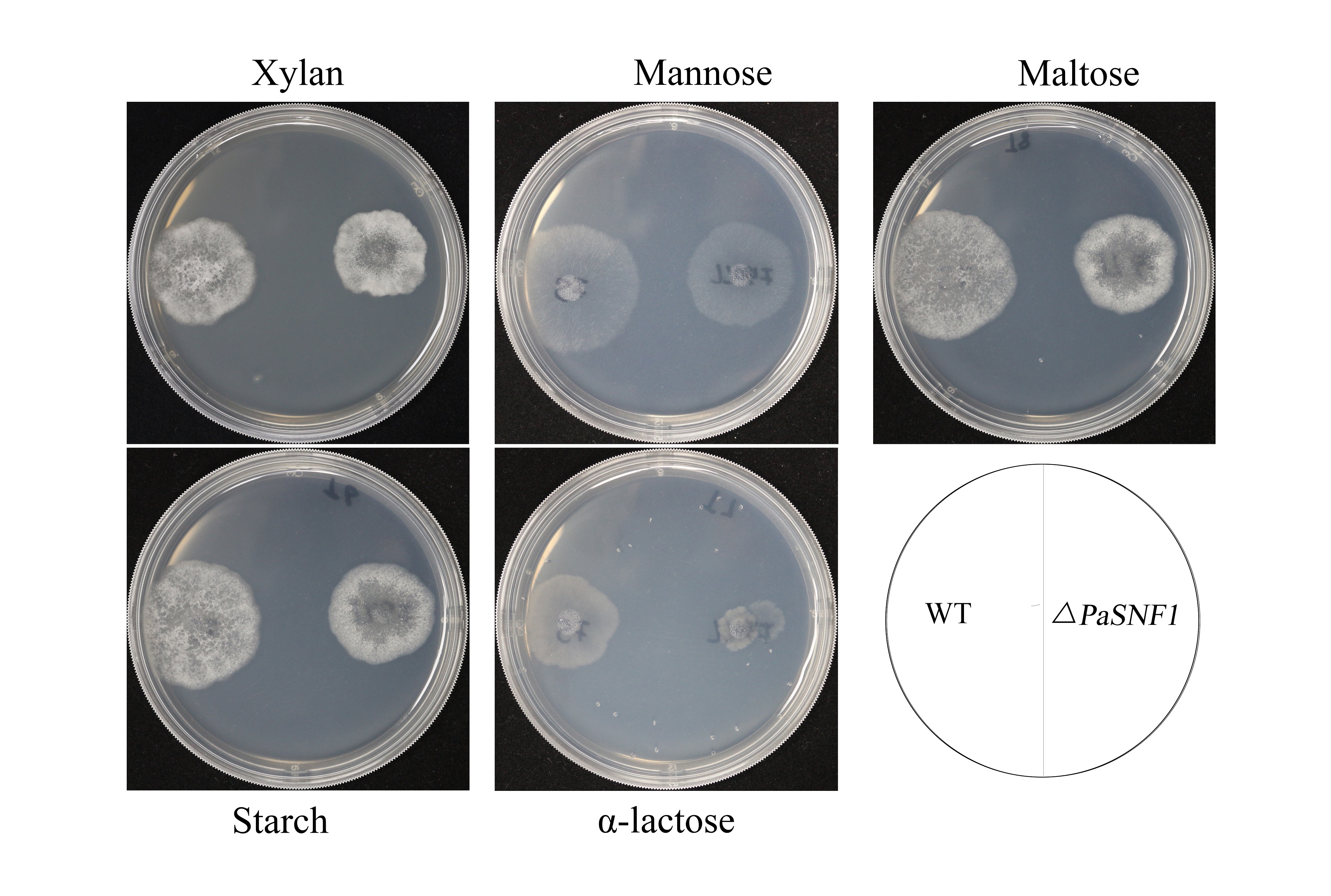

Supplement: Supplementary file 10 [file Image_5.jpg]
